# Supplementary material for: Targeting the 16S rRNA Gene by Reverse Complement PCR Next-Generation Sequencing: Specific and Sensitive Detection and Identification of Microbes Directly in Clinical Samples
Source: Microbiol Spectr. 2023 May 25;11(3):e04483-22. doi: 10.1128/spectrum.04483-22 (PMC10269728; doi:10.1128/spectrum.04483-22)
Supplement: Supplemental file 1 — Supplemental material. Download spectrum.04483-22-s0001.docx, DOCX file, 0.5 MB [file spectrum.04483-22-s0001.docx]

**Supplementary information**

**Supplementary methods**

Samples were tested by the 16S RC-PCR approach and results with 16S Sanger sequencing were compared. Results of the gram stain and culture were reviewed in GLIMS by a clinical microbiologist. Routine culture methods were performed according to the standard operating procedures of the Medical Microbiology Laboratory of the Radboud University Medical Center. Bacterial identification of grown colonies was performed using matrix assisted laser desorption/ionization-time of flight (MALDI-TOF) (Bruker, Billerica, USA). For the extraction of DNA from tissue samples a small piece in saline was added to a tube containing MagNA Lyser Green Beads (Roche) (20s 6500x). A volume of 200 µL was used as input for isolation. Lysis was performed using the MagNA Pure 96 DNA and Viral NA Small Volume Kit (Roche).

16S rRNA Sanger sequencing on clinical samples was performed using the forward primer (5’-AGAGTTTGATCCTGGCTCAG-3’) and the reverse primers (5’-CTTGTGCGGGTCCCCGTCAATTC-3’) and (5’-CTGCGTCCCGTAGGAGTCTGG-3’) (TIB Molbiol) targeting the V1-V2 and V1-V5 domains of the bacterial 16S rRNA gene as described by Weisburg et al. (29). PCR was performed on a Veriti Thermal Cycler (ThermoFisher, Waltham, MA, USA). The following amplification conditions were used: 5 min at 94 ºC, followed by 40 cycles of 1 min at 94 ºC, 1 min at 50 ºC and 2 min at 72 ºC, followed by an incubation of 7 min at 72 ºC. PCR products were separated on a 1.5% agarose gel and visualized with MIDORI Green Advance. Purification of PCR products was performed using the QIAquick Gel Extraction kit (Qiagen). After clean-up the DNA was once more put on gel for quality control to verify efficiency of gel extraction. Additionally, the amount of DNA was quantified by Qubit dsDNA HS kit (ThermoFisher, Waltham, MA, USA) and dependent on the product size a specified concentration was used for Sanger sequencing. Samples were sequenced by the Department of Human Genetics at the Radboudumc using Sanger sequencing. Forward and reverse sequences were aligned using Bionumerics v7.5 (Applied Maths). Sequences were visually inspected and in case of poor quality the sequence was edited. Microorganisms were identified using the web-based basic local alignment tool (BLAST) against the NCBI nucleotide database (nucleotide collection (nr/nt)).

**16S RC-PCR**

RC-PCR was performed using a Veriti Thermal Cycler (ThermoFisher, Waltham, MA, USA). RC-PCR products from pool A and B were individually purified using AmpliClean™ Magnetic Cleanup beads (NimaGen B.V., Nijmegen, The Netherlands) and quantified using the Qubit double strand DNA (dsDNA) High Sensitivity kit on a Qubit 4.0 instrument (ThermoFisher, Waltham, MA, USA). Size distribution was assessed by the TapeStation 2200 (Agilent) using the D1000 High Sensitivity kit (Agilent) according to the manufacturer’s instructions. The library was diluted to 2 nM using low TE buffer. Next-Generation Sequencing (NGS) was performed on an Illumina MiniSeq ® using a MiniSeq high output cartridge for producing 2x150 paired-end sequences (Illumina, San Diego, Ca, USA). Analysis of the data was performed using a custom developed nextflow analysis pipeline called RC-PCR classifier (version 0.2) which is accessible via <https://jordycoolen.github.io/RC-PCR_CLASSIFIER/>. In short, reads are cleaned using fastp (version 0.20.1). Sequence quality control was accessed using multiQC (version 1.12). Identification of bacteria was based on a comparison against the SILVA (138.1 SSURef NR99) database (26) (<https://www.arb-silva.de/>) using *k*-mer alignment (KMA) (version 1.3.28) (30). In Table 1, for samples S6 and S9, for each genus the species with highest coverage is reported. *Moraxella osloensis* and *Xanthomonas* sp. were regarded as clear contaminants and were not reported in Table 2.

**ZymoBIOMICS microbial standards**

Detection of polymicrobial samples was tested by using the ZymoBIOMICS™ Microbial Community DNA Standard (Zymo Research Corporation, CA, USA). Samples were processed according to previously mentioned method of 16S RC-PCR.

For measuring the Limit of Detection (LOD) both ZymoBIOMICS™ Microbial Community Standard II (Log Distribution) and ZymoBIOMICS™ Microbial Community DNA Standard II (Log Distribution) were used. A dilution series was created in triplicate of both products from 0 (undiluted) to -3 (most diluted). Table S2 shows in detail the number of cells present in each dilution series and the amount of DNA and 16S abundance used per sample.

**
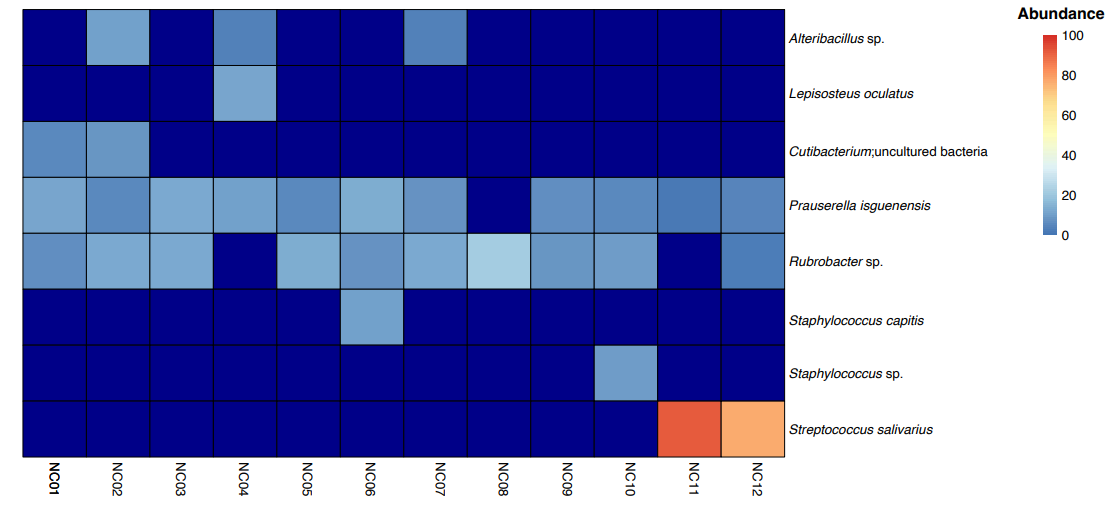
**

**Figure S1. Abundance heatmap of species detected in the negative controls.** Heatmap showing the species identified in the negative control samples. All contaminant bacterial species show a low abundance except for the samples containing *Streptococcus salivarius*. Negative controls contain samples that were processed simultaneously with the clinical samples according to the protocols used for DNA isolation and RC-PCR without adding material.

**Table S1. Identification results of bacterial isolates in triplicate using 16S RC-PCR.**

| **Sample** | **Culture** | **16S RC-PCR (success/replicates)** |
| --- | --- | --- |
| BI 1 | *Neisseria gonorrhoeae* | *Neisseria gonorrhoeae* (3/3) |
| BI 2 | *Klebsiella quasipneumoniae* | *Klebsiella quasipneumoniae* (3/3) |
| BI 3 | *Staphylococcus aureus* | *Staphylococcus aureus* (3/3) |
| BI 4 | *Escherichia coli* | *Escherichia coli* (3/3) |
| BI 5 | *Enterococcus faecium* | *Enterococcus faecium* (3/3) |
| BI 6 | *Haemophilus influenzae* | *Haemophilus influenzae* (3/3) |
| BI 7 | *Bacillus subtilis* | *Bacillus subtilis* (3/3) |
| BI 8 | *Moraxella catarrhalis* | *Moraxella catarrhalis* (3/3) |
| BI 9 | *Bacteroides fragilis* | *Bacteroides fragilis* (3/3) |
| BI 10 | *Clostridium perfingens* | *Clostridium perfingens* (3/3) |
| BI 11 | *Neisseria meningitidis* | *Neisseria meningitidis* (3/3) |

**Figure S2. Results of the Limit of Detection experiment.** 16S RC-PCR results show a comparable 16S abundance according to log distributed microbial community. Based on the dilution series the LOD is between 47.2 – 4.6 cells for *E. coli* with the abundance of 0.069%.

A: 16S abundance of species in Microbial Community DNA Standard II (Log Distribution) B: Amplicons found in species of Microbial Community DNA Standard II (Log Distribution). C: 16S abundance of species in Microbial Community Standard II (Log Distribution). D: Amplicons found in species of Microbial Community Standard II (Log Distribution).

**Table S2. Theoretical composition and Estimated Cell numbers for Limit of Detection experiment.**

|  |  | **Theoretical Composition (%)** | | | **Estimated cell number** | | | | | | | |
| --- | --- | --- | --- | --- | --- | --- | --- | --- | --- | --- | --- | --- |
| **Species** | | **Genomic DNA** | **16S Only** | **Cell Number** | **cell -0** | **cell -1** | **cell -2** | **^cell -3** | **dna -0** | **dna -1** | **dna -2** | **dna -3** |
| *Listeria* | *monocytogenes* | 89.1 | 95.9 | 94.9 | 7592000 | 759200 | 75920 | 7592 | 5694000 | 1138800 | 113880 | 11388 |
| *Pseudomonas* | *aeruginosa* | 8.9 | 2.8 | 4.2 | 336000 | 33600 | 3360 | 336 | 252000 | 50400 | 5040 | 504 |
| *Bacillus* | *subtilis* | 0.89 | 1.2 | 0.7 | 56000 | 5600 | 560 | 56 | 42000 | 8400 | 840 | 84 |
| *Saccharomyces* | *cerevisiae* | 0.89 | NA | 0.12 | 9600 | 960 | 96 | 9.6 | 7200 | 1440 | 144 | 14.4 |
| *Escherichia* | *coli* | 0.089 | 0.069 | 0.058 | 4640 | 464 | 46.4 | 4.64 | 3480 | 696 | 69.6 | 6.96 |
| *Salmonella* | *enterica* | 0.089 | 0.07 | 0.059 | 4720 | 472 | 47.2 | 4.72 | 3540 | 708 | 70.8 | 7.08 |
| *Lactobacillus* | *fermentum* | 0.0089 | 0.012 | 0.015 | 1200 | 120 | 12 | 1.2 | 900 | 180 | 18 | 1.8 |
| *Enterococcus* | *faecalis* | 0.00089 | 0.00067 | 0.001 | 80 | 8 | 0.8 | 0.08 | 60 | 12 | 1.2 | 0.12 |
| *Cryptococcus* | *neoformans* | 0.00089 | NA | 0.00007 | 5.6 | 0.56 | 0.056 | 0.0056 | 4.2 | 0.84 | 0.084 | 0.0084 |
| *Staphylococcus* | *aureus* | 0.000089 | 0.0001 | 0.0001 | 8 | 0.8 | 0.08 | 0.008 | 6 | 1.2* | 0.12 | 0.012 |
|  |  |  |  |  |  | | | |  |  |  |  |

cell: ZymoBIOMICS™ Microbial Community Standard II (Log Distribution)
dna: ZymoBIOMICS™ Microbial Community DNA Standard II (Log Distribution)
NA: Not Applicable
*:is a 1/5 dilution due to limited material

**Table S3. Theoretical composition of Microbial community standard (ZymoBIOMICS**™**) based on genomic DNA.**

| Species | Gram stain | gDNA Abundance (%) |
| --- | --- | --- |
| *Pseudomonas aeruginosa* | - | 12 |
| *Escherichia coli* | - | 12 |
| *Salmonella enterica* | - | 12 |
| *Lactobacillus fermentum* | + | 12 |
| *Enterococcus faecalis* | + | 12 |
| *Staphylococcus aureus* | + | 12 |
| *Listeria monocytogenes* | + | 12 |
| *Bacillus subtilis* | + | 12 |
| *Saccharomyces cerevisiae* | Yeast | 2 |
| *Cryptococcus neoformans* | Yeast | 2 |

**Table S4. Primers in 16S RC-PCR design.**

| Start* | End* | Forward sequence | Reverse sequence | Pool | Target |
| --- | --- | --- | --- | --- | --- |
| 6 | 342 | GAAGAGTTTGATCATGGCTCAG | CTGCTGCCTCCCGTAG | A | V1-2 |
| 334 | 532 | CCAGACTCCTACGGGAGGCAGC | TACCGCGGCTGCTGCTGGCAC | B | V3 |
| 515 | 806 | GTGCCAGCMGCCGCGGTAA | GGACTACHVGGGTWTCTAAT | A | V4 |
| 786 | 939 | GATTAGATACCCTGGTAG | CTTGTGCGGGCCCCCGTCAATTC | B | V5 |
| 961 | 1085 | TCGATGCAACGCGAAGAA | ACATTTCACAACACGAGCTGACGA | A | V6 |
| 1391 | 1541 | TGYACACACCGCCCGTC | AAGGAGGTGATCCANCCYCA | B | V9 |

*Positions are according to16S rRNA of *Escherichia coli* str. K-12 substr. MG1655 NCBI Gene ID 947777.
